# Case Report: From absolute uterine factor infertility (AUI) to motherhood: the crucial role of psychosocial assessment in the first Italian uterus transplantation case

**DOI:** 10.3389/fpsyg.2025.1713821

**Published:** 2026-01-12

**Authors:** Maria Luisa Pistorio, Concetta De Pasquale, Massimiliano Veroux, Alessia Giaquinta, Martina Maria Giambra, Antonino Grasso, Paolo Scollo, Maria Catena Ausilia Quattropani, Pierfrancesco Veroux

**Affiliations:** 1 Vascular Surgery and Organ Transplant Unit, Department of General Surgery and Medical-Surgical Specialties, University Hospital of Catania, Catania, Italy; 2 Department of Educational Sciences, University of Catania, Catania, Italy; 3 Organ Transplant Unit, Department of Surgical and Medical Sciences and Advanced Technologies, University Hospital of Catania, Catania, Italy; 4 Maternal and Child Department, Obstetrics and Gynecology, Cannizzaro Hospital, Catania, Italy

**Keywords:** case report, clinical psychology, infertility, mental health, uterus transplantation

## Abstract

**Introduction:**

Uterus transplantation represents a revolutionary breakthrough for women with absolute infertility of uterine origin, offering them the possibility of realizing the dream of motherhood. This complex procedure has profound medical, ethical, and psychological implications. The aim of our study was to assess the personality, the presence of anxiety and depression, and perceived quality of life in a 29-year-old woman affected by Rokitansky syndrome (MRKH). Furthermore, we hypothesized that projective tests, compared with quantitative assessments, could more easily uncover the deepest, unconscious psychic contents of this woman who has faced such a difficult journey.

**Design:**

Descriptive Longitudinal Study.

**Subjects:**

Single case.

**Main outcome measures:**

The case report follows the Case Reporting (CARE) guidelines. In the pre-transplant evaluation, Giorgia (not her real name) was administered the Machover Test, the Baum Test, the Million Clinical Multiaxial Inventory- III (MCMI-III), the Short Form Health Survey-36 (F-36), the Patient Health Questionnaire-9 (PHQ-9), and the General Anxiety Disorder-7 (GAD-7). The same instruments were re-administered 1 year and 2 years after the birth of her baby girl.

**Results:**

The projective graphic tests highlighted some significant aspects: the facial expression of the female figure became more restless in subsequent evaluations, until aggressiveness emerged (clenched fists) in the last evaluation. The tree was also drawn on a flowerbed in the last evaluations, indicating a sense of loneliness and abandonment.

**Conclusion:**

Overall, uterus transplantation appears to be psychologically well-tolerated, but ongoing evaluation and psychological support are essential.

## 1 Introduction

Infertility, defined as the failure to conceive after 1 year of regular sexual intercourse without the use of contraceptives, often causes significant social distress and is accompanied by numerous psychological and social problems such as depression, anxiety, social isolation, and sexual dysfunction (Sezgin et al., 2016; Boivin et al., 2022). The study by Karpel et al. (2025) found that recipients’ psychological distress due to infertility (FertiQoL) correlated positively with donor anxiety. The emotional aspect of infertility was the most stressful factor for recipients (Karpel et al., 2025).

The awareness of not being able to procreate can lead to a decrease in self-esteem and sense of self-worth. Women who suffer from infertility may feel isolated or stigmatized, especially in cultures where motherhood is highly valued. It is considered a major life crisis and has a strong impact on the quality of life of individuals who experience it (Namdar et al., 2017; Richards et al., 2019; Massarotti et al., 2019).

Uterus transplantation represents a revolutionary breakthrough for women with Absolute Uterine Infertility (AUIF), offering them the possibility of realizing their desire for motherhood. This complex procedure has profound medical, ethical and psychological implications, which can significantly affect the lives of AUIF women. Various studies highlight the importance of psychosocial evaluation in this area (Gerstl et al., 2025; Halkola et al., 2022; Favre-Inhofer et al., 2018).

In the prospective observational study by Järvholm et al. (2020), the psychosocial outcomes of nine women with AUIF and their partners were assessed 2 and 3 years after uterus transplantation. The assessment focused on fertility-related quality of life, mood, and couple relationships. The results showed that transplant failure and failure to achieve parenthood result in psychological strain on couples, which is why psychological support is crucial for recipients and their partners (Wall et al., 2021). In the study by Wall et al. (2021), the psychological impact of infertility was assessed on 21 uterus transplant recipients. Six were pregnant, six had experienced failure and removal of the transplanted uterus, five had given birth to a healthy child and four were awaiting embryo transfer. It emerged that the diagnosis of AUFI had a strong negative impact on psychological well-being, relationships and female identity, and that, on the other hand, the positive impact of uterus transplantation on the healing of the “emotional scars” of AUFI, on female identity and reproductive autonomy was strong (Wall et al., 2021). Motherhood after uterus transplantation reflects a love of life, solidarity and the ability to go beyond one’s fears and anxieties, which undoubtedly shows how strong the couple’s desire for parenthood can be, overcoming all the difficulties such as uterus transplant, immunosuppressive therapy to prevent organ rejection, and homologous assisted fertilization that certainly arise throughout the process necessary to give birth to the much-desired child (Langher et al., 2019; Ribeiro Neto et al., 2025). A review of the literature published up to December 1, 2021 showed a cumulative rate of live births in successful transplant procedures of over 80% in 62 published cases (Brännström et al., 2021). In the study by Järvholm et al. (2021), they sought to understand how women cope with the first years of motherhood after uterus transplantation. The study included seven participants (of which 6 had had AUFI because of congenital absence of the uterus, while 1 had undergone a hysterectomy) who had successfully undergone uterus transplantation and who were given structured interviews once a year for 5 years after transplantation. All 7 participants became pregnant during the study period and became mothers, and it was noted that these women, who seek motherhood after uterus transplantation, generally describe their situation as manageable and comparable to other women who undergo infertility treatments. However, real psychological strains in motherhood after uterus transplantation emerge such as concerns about the health of the child and the effects of immunosuppressants (Järvholm et al., 2021).

While there is no single, universally applicable standard for psychological evaluation for uterine transplantation, international guidelines and recommendations from specialist groups define a mandatory and structured psychological approach for this procedure. Uterus transplantation, being a non-lifesaving and highly complex experimental procedure, requires even greater ethical and psychological rigor than vital organ transplants. International medical and scientific associations, such as the ASRM (American Society for Reproductive Medicine) and the International Society for Uterine Transplantation (ISUTx), have issued clear recommendations that guide clinical centers worldwide. A thorough psychological evaluation of the candidate (recipient) is considered mandatory and must include:

- A full and realistic understanding of the experimental nature of the procedure, the medical risks (including long-term immunosuppressive therapy), and the possibility of failure (organ rejection or pregnancy failure).
- The woman’s ability and willingness to strictly follow the post-transplant treatment regimen (adherence), which is crucial for organ survival and a successful pregnancy.
- Psychological Stability: Screening for anxiety, depression, or other significant psychiatric disorders. Women with absolute uterine factor infertility (AUI), such as those with Mayer-Rokitansky–Küster–Hauser (MRKH) syndrome, may experience significant psychological distress that must be assessed and supported.
- Social Support: Assessment of the family, marital, and social support networks.
- Motivation: Assessment of a realistic and well-balanced motivation, distinguishing between the desire for motherhood and the psychological need to experience pregnancy.

The aim of our study was to assess the personality, the presence of anxiety and depression, and perceived quality of life in a 29-year-old woman affected by Rokitansky syndrome (MRKH), a rare congenital disorder characterized by the absence or incomplete development of the uterus and upper vagina. Furthermore, we hypothesized that projective tests, compared with quantitative assessments, could more easily uncover the deepest, unconscious psychic contents of this woman who has faced such a difficult journey. For these reasons, we used this approach, which we hope will serve as a valuable example for other psychological evaluations of women awaiting uterus transplantation. We hypothesized that the qualitative approach, both through interviews and projective tests, in addition to quantitative assessment, can facilitate the free expression of sensitive and difficult issues, such as those in these cases.

Better understanding the desire for motherhood in women undergoing uterus transplantation will allow us to plan targeted support for this group of patients.

### 1.1 Case history

At the time of the first psychological evaluation, Giorgia was a 29-year-old woman with MRKH Syndrome, diagnosed at age 17. The patient provided written informed consent and permission to use her data before participating in the study. The patient’s confidentiality was rigorously maintained in compliance with the Declaration of Helsinki, and her informed consent for participation in the protocol and for the anonymous publication of this case report was obtained in writing. The Italian Research Project for Uterus Transplantation from a brain-dead donor was approved in 2018 (No. 1438/CNT2018). The project was approved by the Ethics Committee Catania 1 (protocol No. 0026684 on July 3, 2017). In April 2019, Giorgia underwent the psychological evaluation required for inclusion on the waiting list for a uterus transplant. Giorgia has a low level of education (middle school), is a housewife and is married. During the first interviews she appeared well-groomed and had a collaborative attitude. She was well-oriented and used fluent and coherent language. There were no disturbances in the form and content of thought. As regards affective-emotional modulation, her mood appeared to tend towards deflection. She reported that her social relationships were good and also the relationship with her husband. As regards family relationships, they were also serene and supportive. The patient did not meet the diagnostic criteria for any psychiatric disorder according to the DSM-5 criteria (American Psychiatric Association, 2013).

During the clinical psychological interview, Giorgia calmly described her pathology, which was diagnosed at the age of 17: “*At around 15–16 years old, my menstrual cycle had not arrived yet, both my mother and my sister had started at 11 years old; therefore, we suspected that something wasn’t right. So, I had some checks, but everyone told me that everything was fine, and I had to stay calm, that my period would come. Then my mother told me that my grandmother had started at 19 years old, so I could be like her*.” (Original Italian: *“Verso i 15–16 anni, il mio ciclo mestruale non era ancora arrivato, sia mia madre che mia sorella lo avevano avuto a 11 anni; quindi, sospettavamo che qualcosa non andasse. Così, ho fatto alcuni controlli, ma tutti mi dicevano che andava tutto bene, e che dovevo stare tranquilla, che il ciclo sarebbe arrivato. Poi mia madre mi ha detto che mia nonna lo aveva avuto a 19 anni, quindi potevo essere come lei”*).

At the age of 17, Giorgia underwent further medical checks and was diagnosed with MRKH Syndrome. Giorgia said she got married at the age of 21 and that her husband was her salvation: “*My husband arrived in the abyss of my life. When we met, I immediately told him about my syndrome, I said to myself, so that he might run away right away, like my ex-boyfriend did. Instead, he stayed, and we have been together for 12 years. He was not afraid of my syndrome; he always told me: <<I love you just the way you are. Despite his closeness, I spent the nights crying, especially when I found out that some friend or cousin had gotten pregnant: it had become a fixed obsession in my mind*.” (Original Italian: “*Mio marito è arrivato nel baratro della mia vita. Quando ci siamo conosciuti, gli ho subito raccontato della mia sindrome, mi sono detta, perché scappasse subito, come ha fatto il mio ex fidanzato. Invece è rimasto, e stiamo insieme da 12 anni. Non aveva paura della mia sindrome; mi diceva sempre: <<Ti amo così come sei. Nonostante la sua vicinanza, passavo le notti a piangere, soprattutto quando scoprivo che qualche amica o cugina era rimasta incinta: era diventata un’ossessione fissa nella mia mente>>”*).

During a psychological interview, Giorgia, when asked “*Why is a uterus transplant so important to you?* What motivated you to make such a strong choice, which puts your body at risk?,” replied: “*Maybe, it’s not so much the desire to have my own biological child, because, in my opinion, it does not change that much, it’s the experience of pregnancy, other paths would not give me this. I lost my father when I was nine, it was devastating, he had a tumor, he had to fight for his life, but he did not make it, and he suffered a lot. I could fight to give life, why deprive myself of a chance that is given to me? They assured me that I would not risk my life, at worst, my body would reject the uterus. I have never ruled out other paths, surrogate motherhood and adoption, and I will do both regardless of how it goes. If you want, nothing is impossible*.” (Original Italian: *“Forse non è tanto il desiderio di avere un figlio biologico, perché secondo me non cambia poi così tanto, è l’esperienza della gravidanza, altre strade non mi avrebbero dato questo. Ho perso mio padre a nove anni, è stato devastante, aveva un tumore, ha dovuto lottare per la vita, ma non ce l’ha fatta, e ha sofferto molto. Potrei lottare per dare la vita, perché privarmi di una possibilità che mi è stata data? Mi hanno assicurato che non avrei rischiato la vita, al peggio, il mio corpo avrebbe rigettato l’utero. Non ho mai escluso altre strade, maternità surrogata e adozione, e farò entrambe indipendentemente da come andrà. Se vuoi, niente è impossibile”*).

When the possibility of a uterus transplant for Giorgia arose, her husband did not want it, he was afraid for her, for her health, but Giorgia’s motivation was strong, and she was able to convince him. In August 2020, a compatible deceased donor uterus became available at an Italian hospital. Giorgia received a uterus transplant on 08/21/2020, during the COVID-19 pandemic. Giorgia was informed that surgical removal of the received uterus could be performed later, recommended because of medical (i.e., immunosuppression and rejection) or surgical (i.e., cesarean section) complications. After surgery, Giorgia was monitored and evaluated by cervical biopsies, pelvic ultrasound with Doppler of the uterine vessels, and hormone assays every 15 days for the first 3 months and then once a month for the following 3 months. All biopsies showed grade 0 mucosal rejection. This was the first attempted and successful UTx in Italy. In January 2022, Giorgia began her pregnancy after various *in vitro* insemination procedures. The pregnancy progressed regularly, without symptoms or pathologies until week 30, when she tested positive for SARS-CoV-2 Giorgia was admitted to hospital as a precaution, although she did not report symptoms related to the COVID-19 disease. Corticosteroid prophylaxis was performed at the beginning of hospitalization and after 30 days. Finally, Giorgia underwent an emergency cesarean section in the 34th-week. Giorgia gave birth to a female and gave her the name of the donor.

## 2 Materials and methods

The case report follows the Case Reporting (CARE) guidelines (Gagnier et al., 2013).

The following inclusion criteria were met:

- Absence of current or previous serious psychiatric diagnoses (according to DSM-5 criteria) that could compromise compliance or stress management;
- Full and realistic understanding of the experimental nature of the procedure, the short- and long-term medical risks (including immunosuppression), and the possibility of failure;
- Motivation for transplantation and pregnancy judged to be realistic, stable, and not based on maladaptive defense mechanisms such as denial.
- Presence of an adequate and stable social and family support network, essential for support during critical phases (surgery, assisted reproductive technology, high-risk pregnancy).
- Evidence of a life history demonstrating high reliability and the ability to scrupulously adhere to a complex therapeutic regimen.

Having deemed the patient psychologically suitable for the uterus transplant process, a psychodiagnostic evaluation was performed to analyze her personality structure, perceived quality of life, and the possible presence of anxiety and depression.

The patient underwent a psychological evaluation with the administration of several tests at three different times: T0, before the transplant; T1, 1 year after the transplant; and T2, 2 years after the transplant and just after the birth of her daughter. In addition to the quantitative tests, we considered the use of two simple projective tests (which we will explain in more detail below) to facilitate the emergence of deeper, unconscious psychological contents, which patients typically do not easily verbalize with quantitative tests.

In the pre-transplant evaluation (T0), Giorgia was administered the Machover Test (Passi Tognazzo, 1977), the Baum Test (Passi Tognazzo, 1977), the Million Clinical Multiaxial Inventory- III (MCMI-III) (Millon et al., 2008), the Short Form Health Survey-36 (SF-36) (Lins and Carvalho, 2016), the Patient Health Questionnaire (PHQ-9) (Kroenke et al., 1999), and the General Anxiety Disorder-7 (GAD-7) (Spitzer et al., 2006). The same instruments, except the MCMI-III, were re-administered 1 year (T1) and 2 years after the transplant and the birth of her baby (T2).

Throughout the process, in addition to assessments using quantitative and projective tests, the patient underwent supportive psychological sessions (once a month, lasting 45 min each), in which she expressed her fears, difficulties, and hopes related to the uterus transplant and her attempts to conceive. Above all, the projective tests allowed the patient to address her deepest, unconscious thoughts, which were gradually processed appropriately.

### 2.1 Machover test

The Machover, or human figure test, is a projective test that appeals to the spontaneous production of the subject and can be administered to both children and adults. The test is easy to administer, the subject is presented with a sheet of paper, a pencil and an eraser and asked to draw a person or a human figure. The most important thing is to leave the subject free to project. The main aspect of this test is that the drawing of the human figure is the projection of the image of one’s own body and self (Passi Tognazzo, 1977).

### 2.2 Baum test

The Baum Test, or tree drawing, was created by Emil Jucker to arrive at a personality diagnosis and, subsequently, Karl Kock gave this technique an objective validity by publishing a manual in 1949. The tree would be the symbol of man, because of its upright position, and tells us something about the subject’s personality. The subject is presented with a sheet of paper and a pencil and asked to draw a fruit tree (Passi Tognazzo, 1977).

### 2.3 Million clinical multiaxial inventory-III (MCMI-III)

The MCMI-III is a personality test that considers a manifestation of the entire complexity of the person with a symptomatology that manifests itself in numerous clinical areas. It is composed of 175 items and 24 clinical scales, a very basic vocabulary is used, corresponding to that of the third year of middle school, and the duration is approximately 20–30 min (Millon et al., 2008).

### 2.4 Short form health Survey-36 (SF-36)

The SF-36 is a very popular questionnaire for the assessment of health-related quality of life. The SF-36 includes 8 scales: physical functioning (PF), role physical (RP), bodily pain (BP), general health (GH), vitality (VT), social functioning (SF), role emotional (RE) and mental health (MH). From these scales, two dimensions can be derived: one regarding physical health and one regarding mental health (Lins and Carvalho, 2016).

### 2.5 Patient health questionnaire-9 (PHQ-9)

The PHQ-9 is a test for the evaluation of depression according to the criteria of the Diagnostic and Statistical Manual of Psychiatry (DSM-V) (American Psychiatric Association, 2013), in which the patient is asked if he/she has experienced any depressive symptoms in the previous two weeks. It is composed of 9 items on a Likert scale ranging from 0 “never” to 3 “almost every day” (Kroenke et al., 1999).

### 2.6 General anxiety disorder-7 (GAD-7)

The GAD-7 is a test that has the function of evaluating whether there are anxious tendencies according to the criteria of the Diagnostic and Statistical Manual of Psychiatry (DSM-V) (Wall et al., 2021), in which the patient is asked if he/she has experienced any anxious symptoms in the previous two weeks. It is composed of 7 items on a Likert scale ranging from 0 “never” to 3 “almost every day” (Spitzer et al., 2006).

## 3 Results

Regarding the study of personality, at time T0, the administration of the MCMI-III highlighted obsessive-compulsive (scale 7 = 87), histrionic (scale 4 = 100) and narcissistic (scale 5 = 70) personality patterns (Table 1). According to the reference manual, the profile is considered normal because, in the absence of a specific pathology, it is precisely the absence of pathology that causes these three scales to rise. Therefore, the elevation of the Narcissistic, Histrionic, and Obsessive-Compulsive scales reflects healthy personality characteristics (Millon et al., 2008).

**Table 1:** Psychological test scores over time.

MCMI-III	T0	T1	T2
Disclosure	22		
Desirability	84		
Debasement	0		
Schizoid	27		
Avoidant	7		
Depressive	7		
Dependent	15		
Histrionic	**100**		
Narcissistic	**70**		
Antisocial	7		
Sadistic	7		
Compulsive	**87**		
Negativistic	19		
Masochistic	7		
Schizotypal	4		
Borderline	4		
Paranoid	16		
Anxiety	4		
Somatoform	4		
Bipolar, Manic	28		
Dysthymia	4		
Alcohol dependence	4		
Drug dependence	4		
Posttraumatic stress disorder	4		
Thought disorder	4		
Major depression	4		
Delusional disorder	28		
**GAD-7**	4	3	3
**PHQ-9**	4	3	3
**SF-36**			
Physical functioning	100	100	60
Role physical	50	100	100
Bodily pain	64	100	100
General health	95	70	56
Vitality	100	90	85
Social functioning	87	37	87
Role emotional	100	100	100
Mental health	92	84	92

Values in bold: values above the cut-off.

**Figure 1:**
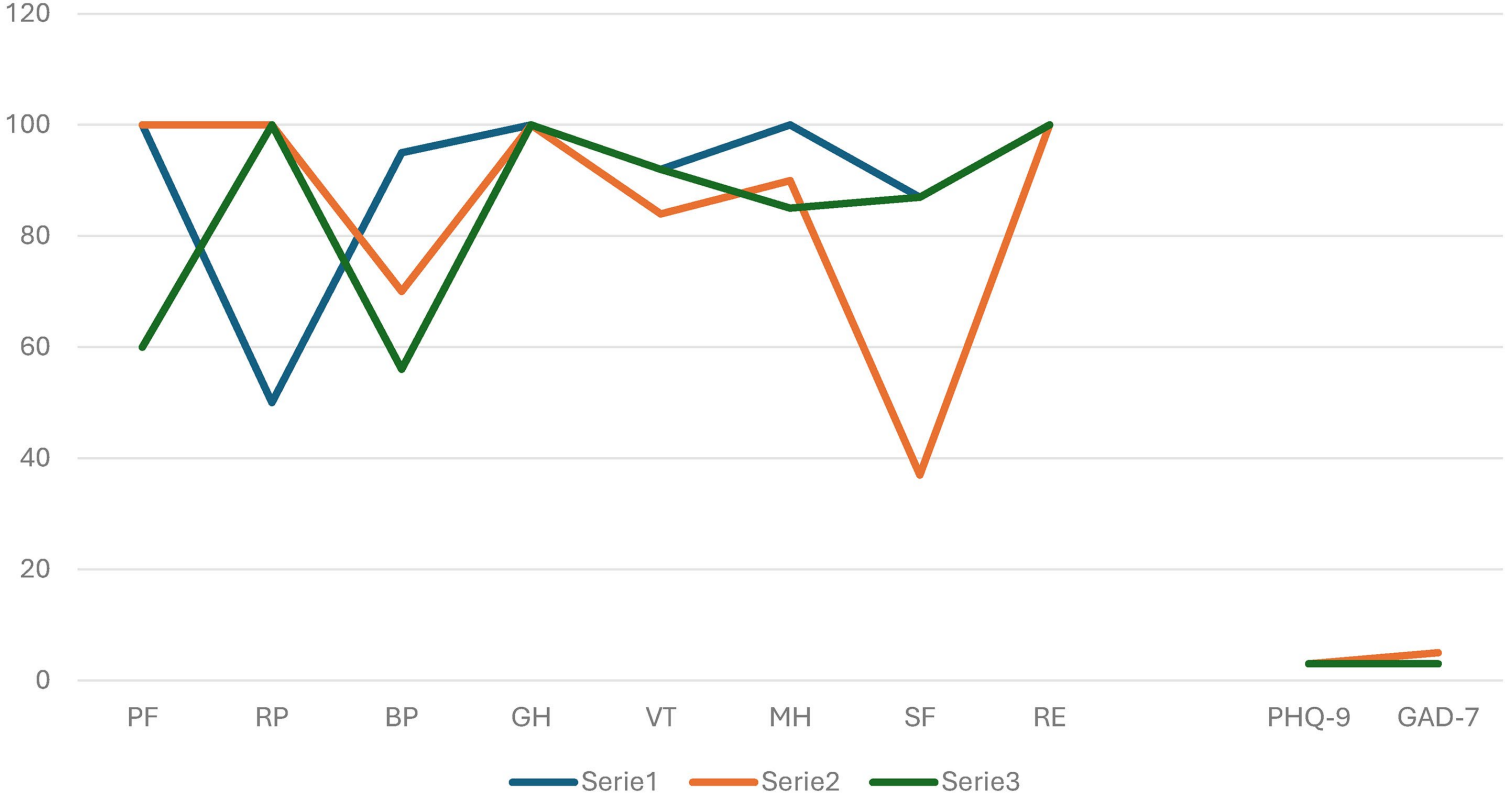
Anxiety, depression, and quality of life symptoms at the three administration times T0, T1, and T2.

Regarding the patient’s psychodiagnostic evaluation through quantitative tests, no significant changes were highlighted in the results related to the three administration times (T0, T1, T2), see Figure 1 and Table 1.

In fact, the quality of life always remained adequate. Only social functioning underwent a decrease in the period of lockdown probably caused by the COVID-19 pandemic, during which Giorgia reported having greatly reduced all interpersonal relationships. While the dimensions related to physical health (role physical and general health) slightly reduced during the last months of pregnancy, a period in which Giorgia reported feeling more tired and fatigued than usual. The GAD-7 and PHQ-9 also did not highlight the presence of anxiety and depression at any of the three assessment times (Table 1).

**Figure 2:**
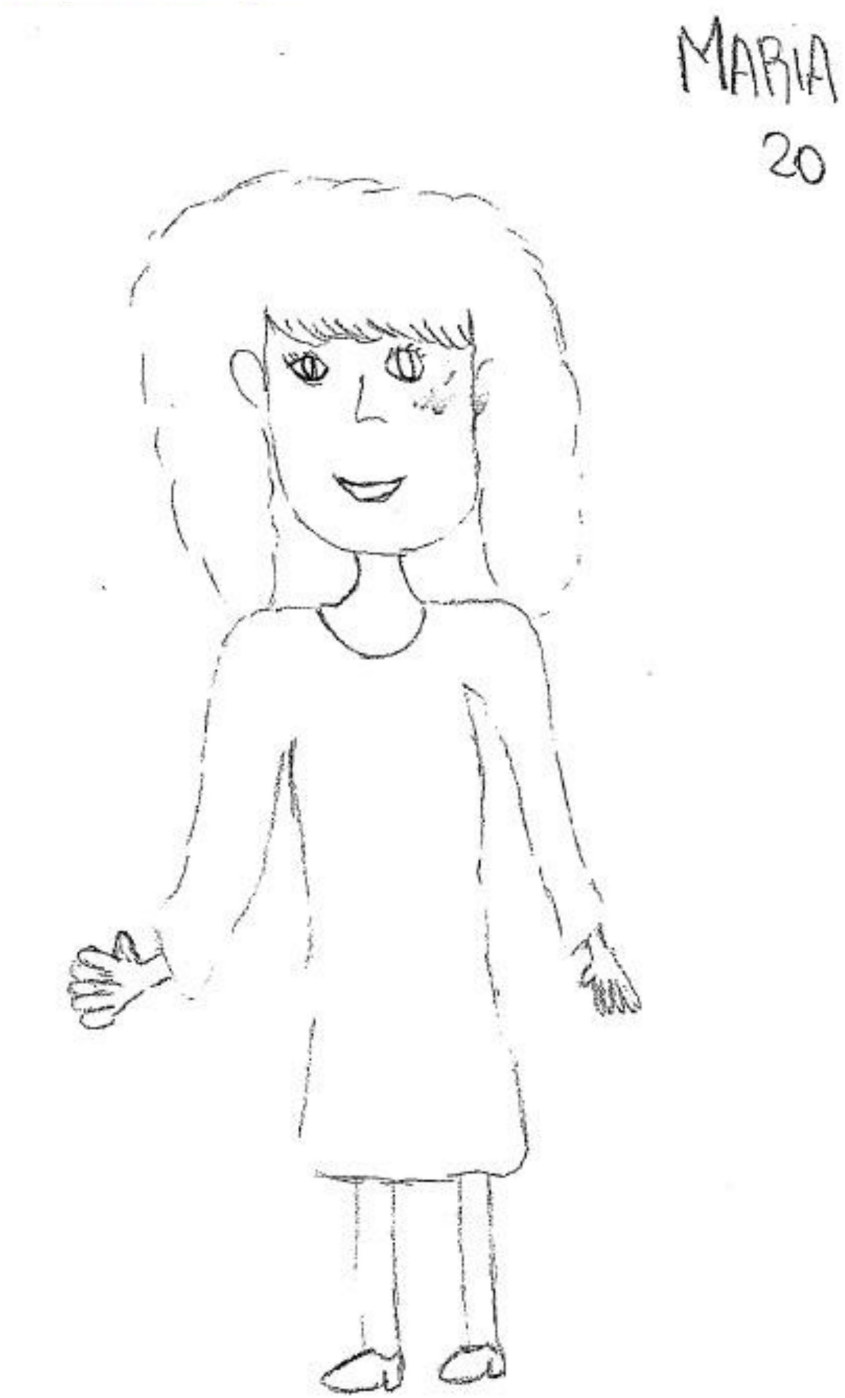
Pre-transplant female figure.

The results of the projective graphic tests (Machover test and Baum test), reveal different and significant results in the three evaluation times:

In the pre-transplant assessment, regressive tendencies, ambivalence between insecurity and impulsivity, and a need for protection emerged (see Figure 2).

In the assessment 1 year after the transplant (T1), attachment to the past and to her mother, and feelings of loneliness and abandonment emerged.

**Figure 3:**
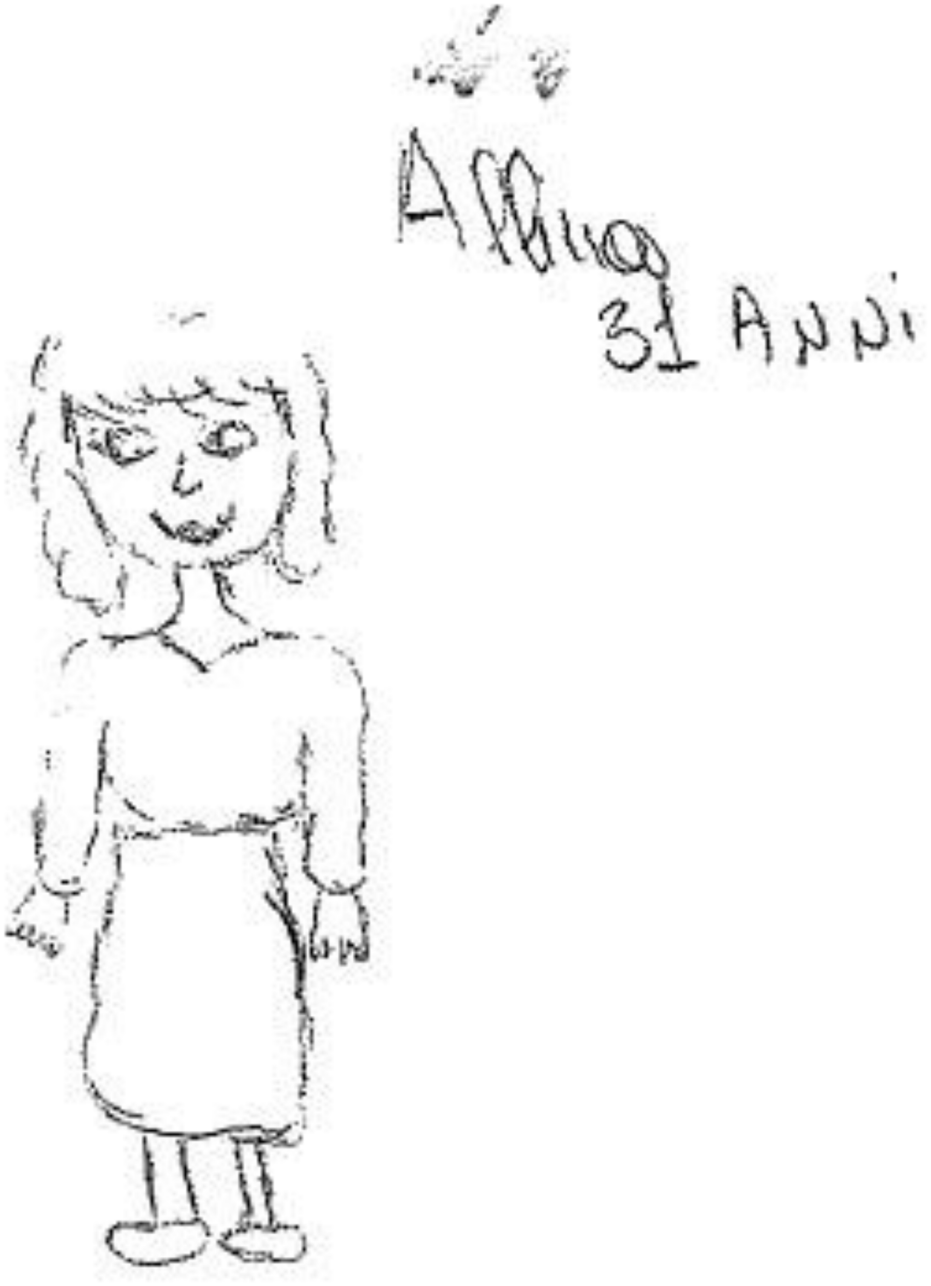
Female figure post-transplant and maternity.

In the assessment 2 years after the transplant and maternity (T2), anxiety, depression, and a need for protection emerged; attachment to the past and to her mother, and feelings of loneliness and abandonment were confirmed (see Figure 3).

From the analysis of the projective graphic test results, we selected the figures that seemed most significant for the changes that emerged.

## 4 Discussion

The concept of gaining childbearing ability via uterus transplantation motivates many infertile women to pursue giving birth to their own children. However, challenges exist regarding donor, recipient, and fetus. Both women and children of uterus transplantation need special consideration because of prematurity-related neonatal problems and the long-term effects of transplant pregnancy (Ongun et al., 2024).

According to the review by Ribeiro Neto et al. (2025), therapeutic decisions are permeated by emotional and ethical complexity, influencing the entire trajectory from the initial choice to the various available options. In response to the challenges of treatment, anxiety and depression commonly manifest, involving not only the patients but also their relational networks.

Although uterus transplantation seems to have a positive effect on self-image there is a lack of knowledge about how women who have received uterine grafts experience pregnancy attempts, pregnancy itself and the first years of motherhood. There are real psychological strains in motherhood after uterus transplantation, such as the concern the women express relating to the health of the child and the effects of immunosuppressants (Veroux et al., 2024). This aspect did not seem to have disturbed Giorgia, who, although aware of the risks, decided to go ahead, since her desire for motherhood was stronger than everything else. Regarding psychosocial outcomes there are many potential stressful events for motherhood after uterus transplantation and these events may have an impact on quality-of-life and mental well-being. Adjusting to a new role as a parent after a uterus transplant can require significant adjustment and may lead to identity conflicts (Järvholm et al., 2024). In our case, for example, with the role of parent Giorgia discovers a difficult and complicated relationship with her mother, in which her expectations regarding a grandmother present with her daughter and granddaughter will be disappointed. The patient reported that her mother was more present during the period in which the transplant path was being attempted. The projective graphic tests highlighted some significant aspects that changed over time: the facial expression of the female figure became more restless in subsequent evaluations, until aggressiveness emerged (clenched fists) in the last evaluation. The tree was also drawn on a flowerbed in the last evaluation, indicating a sense of loneliness and abandonment. The changes observed in the projective tests (T1/T2), particularly the emergence of aggression and loneliness, do not indicate psychopathological destabilization but reflect the psychological costs of adaptation to a high-pressure experimental pathway. Loneliness is likely correlated with the prolonged isolation and medical control post-transplantation and the emotional burden of being a clinical pioneer. Aggression, conversely, may represent the figurative expression of frustration over the loss of autonomy and the constant struggle for graft integration and maintaining the pregnancy under the threat of rejection.

The feeling of loneliness and abandonment, which appeared during pregnancy and remained even after the birth of the child, could also be linked to the dissatisfaction that Giorgia expressed regarding the relationship with her mother. Giorgia reported how her mother was a grandmother who was not very present with her and her granddaughter: “she does not help me with anything, she only thinks about herself”. (Original Italian: “lei non mi aiuta in niente, pensa solo a se stessa”).

Regarding health-related quality-of-life, however, no significant changes were highlighted in the results at the three evaluation times. There were no limitations in the emotional role. Only social activities decreased during the lockdown period, probably caused by the COVID-19 pandemic, during which Giorgia reported having reduced all her interpersonal relationships. Overall, the strong desire for motherhood and the assumption of the new role of mother after a long period of infertility required Giorgia to make a significant psychological adjustment, which was made possible by the continuous psychological support received, from pre-transplant to post-transplant and even after the birth of her child.

The Dallas Uterus Transplant Study reported a 55% live birth rate for attempted transplants and 79% for technically successful transplants. However, this procedure is not without its challenges (Johannesson et al., 2021; Järvholm et al., 2015). Studies have shown that uterus transplantation recipients and their partners generally exhibit psychological stability before and after the procedure, with scores similar to or better than relevant norm groups (Järvholm and Warren, 2022). However, recipients may experience psychological issues including adapting to life with a transplanted uterus, compliance with immunosuppressive treatment, and managing expectations throughout the process (Pittman et al., 2020). Overall, uterus transplantation appears to be psychologically well-tolerated, but ongoing evaluation and psychological support are essential. As uterus transplantation programs develop worldwide, they aim to meet the demands of well-informed patients seeking this innovative fertility treatment (Järvholm et al., 2024; Pittman et al., 2020).

Our case study highlights the importance, in addition to standard quantitative assessments, of combining them with projective tests. These tests allow for greater freedom of expression, addressing deeper, unconscious issues that are often denied or minimized in quantitative test responses. In this case, for example, the patient did not reveal any emotional difficulties in navigating her journey, but by exploring the findings from the projective graphic tests in psychological interviews, she was able to more easily discuss her fears, difficulties, disappointments, and so on, thus allowing for adequate processing of her experiences.

Our recommendation, therefore, is not to limit ourselves to standard quantitative assessments of these patients, but to delve deeper into their more profound issues, which are difficult to openly verbalize.

### 4.1 Limitations and future research

We first have to restate that this was the very first UTx transplantation in Italy, with only about 80 cases worldwide to date. Although our case report demonstrates the feasibility of pregnancy after uterine transplantation, it is essential to recognize the limitations inherent in a single clinical case study. The generalizability of psychological and clinical data is inherently limited, as the experience and resilience of a single patient are not necessarily representative of the broader population of women with AUI. Future research should focus on expanding the sample size through multicenter studies and implementing prospective longitudinal studies with larger sample sizes. Such studies are necessary to standardize psychosocial screening protocols, validate predictors of success such as compliance and psychological adjustment, and define the long-term impact of immunosuppressive therapy on recipients’ mental health and quality of life.

## 5 Conclusion

Uterus transplantation has emerged as a promising option for women with absolute uterine factor infertility to achieve genetic and gestational motherhood. Nonetheless, the deeper psychological aspects of these women undergoing a similar process must be carefully evaluated. Further studies that also explore these patients’ experiences from a more qualitative perspective would be desirable.

## Data Availability

The raw data supporting the conclusions of this article will be made available by the authors, without undue reservation.

## References

[ref1] American Psychiatric Association (2013). Diagnostic and Statistical Manual of Mental Disorders: DSM-5. 5th Edn. Washington, D.C.: American Psychiatric Publishing.

[ref2] Boivin J. Vassena R. Costa M. Vegni E. Dixon M. Collura B. . (2022). Tailored support may reduce mental and relational impact of infertility on infertile patients and partners. Reprod. Biomed. Online 44, 1045–1054. doi: 10.1016/j.rbmo.2022.01.015, 35351377

[ref3] Brännström M. Belfort M. A. Ayoubi J. M. (2021). Uterus transplantation worldwide: clinical activities and outcomes. Curr. Opin. Organ Transplant. 26, 616–626. doi: 10.1097/MOT.0000000000000936. 34636769, 34636769

[ref4] Favre-Inhofer A. Rafii A. Carbonnel M. Revaux A. Ayoubi J. M. (2018). Uterine transplantation: review in human research. J. Gynecol. Obstet. Hum. Reprod. 47, 213–221. doi: 10.1016/j.jogoh.2018.03.006, 29574054

[ref5] Gagnier J. J. Kienle G. Altman D. G. Moher D. Sox H. Riley D. (2013). The CARE guidelines: consensus-based clinical case report guideline development. J. Diet. Suppl. 10, 381–390. doi: 10.3109/19390211.2013.830679, 24237192

[ref6] Gerstl B. Kehag E. Mallinder H. Baker T. Arulpragasam K. David C. . (2025). Psychological and emotional profiles of Australian uterine transplant potential recipients: a comparison with international trials. Acta Obstet. Gynecol. Scand. 104, 528–539. doi: 10.1111/aogs.14974., 39324432 PMC11871097

[ref7] Halkola S. T. Koivula M. Aho A. L. (2022). A qualitative study of the factors that help the coping of infertile women. Nurs. Open 9, 299–308. doi: 10.1002/nop2.1062, 34581511 PMC8685867

[ref8] Järvholm S. Bokström H. Enskog A. Hammarling C. Dahm-Kähler P. Brännström M. (2021). Striving for motherhood after uterus transplantation: a qualitative study concerning pregnancy attempts, and the first years of parenthood after transplantation. Hum. Reprod. 37, 274–283. doi: 10.1093/humrep/deab260, 34865019

[ref9] Järvholm S. Dahm-Kähler P. Kvarnström N. Brännström M. (2020). Psychosocial outcomes of uterine transplant recipients and partners up to 3 years after transplantation: results from the Swedish trial. Fertil. Steril. 114, 407–415. doi: 10.1016/j.fertnstert.2020.03.043, 32709381

[ref10] Järvholm S. Johannesson L. Clarke A. Brännström M. (2015). Uterus transplantation trial: psychological evaluation of recipients and partners during the post-transplantation year. Fertil. Steril. 104, 1010–1015. doi: 10.1016/j.fertnstert.2015.06.038. 26231464, 26231464

[ref11] Järvholm S. Kättström A. Kvarnström N. Dahm-Kähler P. Brännström M. (2024). Long-term health-related quality-of-life and psychosocial outcomes after uterus transplantation: a 5-year follow-up of donors and recipients. Hum. Reprod. 39, 374–381. doi: 10.1093/humrep/dead245, 37995381 PMC10833084

[ref12] Järvholm S. Warren A. M. (2022). Uterus transplantation: lessons learned from a psychological perspective. Clin. Obstet. Gynecol. 65, 52–58. doi: 10.1097/GRF.0000000000000673. 35045025, 35045025

[ref13] Johannesson L. Testa G. Putman J. M. McKenna G. J. Koon E. C. York J. R. . (2021). Twelve live births after uterus transplantation in the Dallas UtErus transplant study. Obstet. Gynecol. 137, 241–249. doi: 10.1097/AOG.0000000000004244, 33416285

[ref14] Karpel L. Nicaise M. Carbonnel M. Le Marchand M. Racowsky C. Pirtea P. . (2025). Psychological evaluation of candidates for the uterus transplantation French trial. Acta Obstet. Gynecol. Scand. 104, 522–527. doi: 10.1111/aogs.15004, 39737538 PMC11871084

[ref15] Kroenke K. Spitzer R. L. Williams J. B. W. 1999 Patient Health Questionnaire-9 (PHQ-9)

[ref16] Langher V. Fedele F. Caputo A. Marchini F. Aragona C. (2019). Extreme desire for motherhood: analysis of narratives from women undergoing assisted reproductive technology (ART). Eur. J. Psychol. 15, 292–311. doi: 10.5964/ejop.v15i2.1736, 33574956 PMC7871747

[ref17] Lins L. Carvalho F. M. (2016). SF-36 total score as a single measure of health-related quality of life: scoping review. SAGE Open Med. 4:205031211667172. doi: 10.1177/2050312116671725, 27757230 PMC5052926

[ref18] Massarotti C. Gentile G. Ferreccio C. Scaruffi P. Remorgida V. Anserini P. (2019). Impact of infertility and infertility treatments on quality of life and levels of anxiety and depression in women undergoing in vitro fertilization. Gynecol. Endocrinol. 35, 485–489. doi: 10.1080/09513590.2018.1540575, 30612477

[ref19] Millon T. . (2008). MCMI-III Millon Clinical Multiaxial Inventory-III. 2rd Edn: Giunti O.S. Organizzazioni Speciali.

[ref20] Namdar A. Naghizadeh M. M. Zamani M. Yaghmaei F. Sameni M. H. (2017). Quality of life and general health of infertile women. Health Qual. Life Outcomes 15:139. doi: 10.1186/s12955-017-0712-y, 28701163 PMC5508693

[ref21] Ongun H. Celik K. Arayici S. Dogan N. U. Mendilcioglu I. Ozkan O. . (2024). Miracles of science: birth after uterus transplantation. J. Obstet. Gynaecol. Res. 50, 5–14. doi: 10.1111/jog.15825, 37922953

[ref22] Passi Tognazzo D. (1977). Metodi e tecniche nella diagnosi della personalità. 2rd Edn. Firenze: Giunti Barbera.

[ref23] Pittman J. Mogensen L. Brännström M. Chan W. Morrison N. (2020). Uterus transplantation: perspectives of Australian women with absolute uterine factor infertility regarding desirability and utility. Aust. N. Z. J. Obstet. Gynaecol. 60, 264–270. doi: 10.1111/ajo.13114, 31916256

[ref24] Ribeiro Neto B. Barreiro M. Tomé A. Vale-Fernandes E. (2025). Psychosocial aspects of infertility and the impact of assisted reproductive techniques - a comprehensive review. JBRA Assist. Reprod. 29, 378–393. doi: 10.5935/1518-0557.20250002, 40305398 PMC12225119

[ref25] Richards E. G. Agatisa P. K. Davis A. C. Flyckt R. Mabel H. Falcone T. . (2019). Framing the diagnosis and treatment of absolute uterine factor infertility: insights from in-depth interviews with uterus transplant trial participants. AJOB Empir. Bioeth. 10, 23–35. doi: 10.1080/23294515.2019.1572672. 30855220, 30855220

[ref26] Sezgin H. Hocaoglu C. Guvendag-Guven E. S. (2016). Disability, psychiatric symptoms, and quality of life in infertile women: a cross-sectional study in Turkey. Shanghai Arch. Psychiatry 28, 86–94. 27605864 10.11919/j.issn.1002-0829.216014 PMC5004092

[ref27] Spitzer R. L. Kroenke K. Williams J. B. W. Löwe B. 2006 Generalized Anxiety Disorder 7 (GAD-7) 10.1001/archinte.166.10.1092 16717171

[ref28] Veroux P. Scollo P. Giaquinta A. Roscitano G. Giambra M. M. Pecorino B. . (2024). Uterus transplantation from deceased donors: first Italian experience. J. Clin. Med. 13:6821. doi: 10.3390/jcm13226821, 39597965 PMC11594497

[ref29] Wall A. Johannesson L. Sok M. Warren A. M. Gordon E. J. Testa G. (2021). The journey from infertility to uterus transplantation: a qualitative study of the perspectives of participants in the Dallas uterus transplant study. BJOG 129, 1095–1102. doi: 10.1111/1471-0528.17052, 34889028

